# Outcomes of and factors influencing the arthroscopic treatment of rotator cuff injury with the patient in the lateral-lying and beach chair positions

**DOI:** 10.1097/MD.0000000000025797

**Published:** 2021-05-07

**Authors:** Minghua Zhang, Daohua Chen, Rong Wu, Dongfeng Chen, Jiajing Lai

**Affiliations:** Department of Orthopedics and Traumatology, Longyan First Hospital Affiliated with Fujian Medical University, Long Yan, Fujian, China.

**Keywords:** arthroscopy, beach chair position, lateral-lying position, rotator cuff injury

## Abstract

To compare the postoperative effects of arthroscopy for rotator cuff injury with patients in the lateral-lying position (LLP) and beach chair position (BCP), and to identify factors influencing these effects.

Data from patients with rotator cuff injuries who underwent shoulder arthroscopy in the LLP (n = 115, 53.24%) or BCP (n = 101, 46.76%) between January 2013 and 2016 and were followed for >3 years were analyzed. The American Shoulder and Elbow Surgeons shoulder score, University of California at Los Angeles shoulder score (UCLASS), and visual analog scale (VAS) score were used to evaluate patients’ shoulder function and pain preoperatively and at the last follow-up examination. The abduction and lateral rotation angles were measured. The influences of patient characteristics were compared between the LLP and BCP subgroups defined by UCLASSs (excellent, good, acceptable, poor).

Postoperative injury characteristics, UCLASSs, and VAS scores were better in the LLP group than in the BCP group (all *P* < .05). Among patients with good UCLASSs, preoperative pain duration was longer in the LLP group than in the BCP group (*P* < .05); among those with acceptable UCLASSs, this duration was longer in the BCP group than in the LLP group (*P* < .05). The preoperative flexion angle differed between groups (*P* < .05). Among patients with excellent and good UCLASSs, the postoperative external rotation angle was greater in the LLP group than in the BCP group (*P* < .05). The LLP group contained more excellent UCLASSs than did the BCP group (*P* < .05). It also contained more small, medium, and large tear cases than did the BCP group (all *P* < .05).

The effect of arthroscopy for rotator cuff injury was better when the operation was performed with the patient in the LLP. Either position is suitable for the arthroscopic treatment of partial rotator cuff tears. The LLP is more suitable in cases of small and medium-sized tears and those with large preoperative lateral rotation angles. The BCP should be used for patients with large preoperative flexion angles.

## 1 Introduction

Shoulder arthroscopy has several advantages over open surgery, such as the provision of a more comprehensive view of intra-articular pathologies, reduced morbidity due to the ability to use smaller incisions, and the potential for more rapid rehabilitation and return to work.^[1]^ It is used commonly in the diagnosis and treatment of shoulder conditions, such as rotator cuff injuries, labral tears, proximal biceps pathologies, loose bodies, degenerative arthritis, adhesive capsulitis, subacromial impingement, calcified supraspinatus tendinitis, and bone defects in recurrent anterior shoulder dislocation. Shoulder arthroscopy is the second most commonly performed orthopedic procedure, after knee arthroscopy for partial meniscectomy.^[2]^

Rotator cuff injury is the most common shoulder pathology, representing >60% of shoulder injuries.^[3]^ Pain and limited function are its main symptoms.^[4]^ About 35,000 to 75,000 patients with rotator cuff injuries undergo rotator cuff repair surgery in the United States annually.^[5]^ Shoulder arthroscopy is currently performed with the patient in the lateral-lying position (LLP) or beach chair position (BCP); the choice of position is of great importance, as it affects the prognosis. Various scoring systems, such as the American Shoulder and Elbow Surgeons (ASES) shoulder score,^[6]^ the University of California at Los Angeles shoulder score (UCLASS),^[7]^ and visual analog scales (VASs), are used to assess the outcomes of shoulder arthroscopy performed using the 2 patient positions, but evidence for which position is more advantageous remains inconclusive.^[8]^ This retrospective analysis was conducted to investigate the clinical efficacy and relative advantages of arthroscopic rotator cuff repair surgeries performed with patients in the LLP and BCP using the ASES score, UCLASS, and VAS score, as well as measures of flexion, abduction, and the body lateral rotation angle.

## 2 Materials and methods

### 2.1 Patient selection and injury classification

In total, 252 patients who underwent shoulder arthroscopy for rotator cuff repair in the Department of Orthopedics and Traumatology, Longyan First Hospital Affiliated with Fujian Medical University, between January 2013 and January 2016 were screened for inclusion in this study. The inclusion criteria were: admission with rotator cuff injury, >3 years of follow up, and completeness and availability of preoperative and postoperative clinical scores and imaging data. Exclusion criteria were: accompanying shoulder neurovascular injury, accompanying shoulder dislocation, previous history of shoulder surgery, and noncompletion of rehabilitation training. Data from 216 patients were included in the analysis. Of them, 115 patients (53.24% 65 men, 50 women) with a mean age of 63.4 ± 12.2 years (range, 29–82 years) underwent surgery on the left (n = 63) or right (n = 52) shoulder in the LLP. One hundred one patients (46.76% 58 men, 43 women) with a mean age of 62.1 ± 13.4 years (range, 30–81 years) underwent surgery on the left (n = 62) or right (n = 39) shoulder in the BCP.

Preoperatively, medical histories and symptom reports were taken and all patients underwent physical and imaging (orthotopic shoulder radiography, supraspinatus exit radiography, and shoulder magnetic resonance imaging [MRI]) examinations for the diagnosis of rotator cuff injury. According to the Gerber classification,^[9]^ the LLP group comprised 23 cases of partial rotator cuff injury, 72 cases of small or medium-sized rotator cuff injury, and 20 cases of huge rotator cuff injury; the BCP group comprised 22 cases of rotator cuff injury, 61 cases of small or medium-sized cuff sleeve injury, and 18 cases of huge rotator cuff injury. Of the 216 patients, 179 underwent initial conservative treatment, for an average of 4 ± 1.2 months (range, 1–13 months), and decided to undergo surgical treatment after their symptoms had not improved. Thirty-seven patients had acute trauma or short-term (1 week–1 month) symptoms. Arthroscopic surgery was performed when aggravation persisted after 1 month of conservative treatment. All sleeves were repaired intraoperatively with 4.5-mm absorbable thread anchors (Arthrex Products, FL, USA). All patients provided informed consent to study participation. The study was authorized by the hospital's ethics committee (no. 2020065).

### 2.2 Surgical method

**Figure 1:**
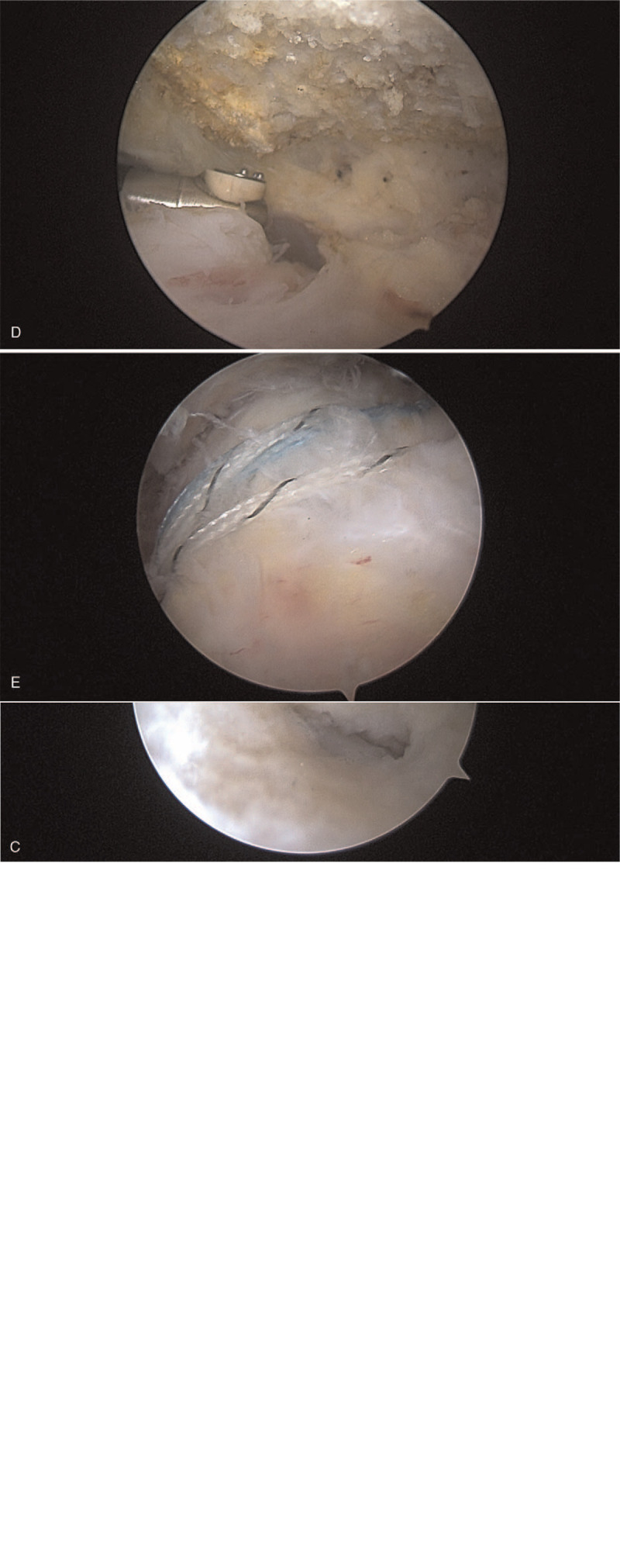
A: Lateral lying position. B: Beach chair position. C: Large rotator cuff injury. D: Acromion. E: Rotator cuff repair.

The same 3-person surgical team performed all surgeries. After the induction of anesthesia, the patient was placed in the LLP with the affected limb under skin traction (Fig. 1A) or in the BCP without traction (Fig. 1B). The affected shoulder and upper limbs were routinely disinfected with iodophor and a sterile towel, the connected to a shoulder arthroscopic surgical system (Arthrex Products). The shoulder joint was punctured, 40 mL saline was injected, and the arthroscopic instruments were introduced using the commonly used posterior, anterior, or lateral approach and arthroscopy was performed. From the intra-articular synovium, glenoid lip, articular cartilage, glenohumeral ligament, biceps tendon, and rotator cuff, rupture of the rotator cuff supraspinatus muscle was visualized (Fig. 1C). Then, a plasma knife and electric planer were used to clean the subacromion. The acromial impact was determined by direct visual observation. Acromioplasty was performed, depending on the shape of the acromion and the impact on performance beneath it (Fig. 1D). Under the shoulder peak, the loosened cellulose bands, inflamed bursa tissue, and supraspinatus crevices were cleared. Under direct visualization, the residual tissue at the stop point of the rotator cuff was removed to freshen this point. The inner and outer rows of fixing screws were inserted with wire anchors, and double rows of sutures were used to complete the rotator cuff tear repair. The tear was inspected to confirm that the repair was solid and stable (Fig. 1E), a large volume of physiological saline was used to lavage the joint cavity, the incision was closed, and protected the shoulder joint with a shoulder brace. The operation was successful (∼5 mL intraoperative bleeding, no specimen), and the patient was returned to the ward.

### 2.3 Evaluation of surgical efficacy

The ASES, UCLASS, and VAS were used to evaluate the efficacy of the arthroscopic surgeries. The ASES is used to assess patients’ subjective pain and life function (50 points each; total possible score = 100). The UCLASS comprises 10 points each for shoulder pain and function, and 5 points each for active flexion activity, flexion strength, and patients’ subjective satisfaction. The maximum score is 35 points; 34 and 35 points indicate an excellent outcome, 28 to 33 points indicate a good outcome, 21 to 27 points indicate an acceptable outcome, and ≤20 points indicate a poor outcome. A VAS ranging from 0 (no pain) to 10 (severe pain) also was used to assess patients’ subjective pain. The angle of abduction and lateral rotation of the affected shoulder were measured. All assessments were performed preoperatively and at the last follow-up visit.

### 2.4 Postoperative rehabilitation exercise

Patients wore 45° shoulder abductor braces 24 hours a day for 12 weeks after surgery. They initiated hand-opening exercises (500/d) on the day after surgery. Three to 5 days after surgery, elbow flexion, and extension and forearm rotation with the support of the healthy hand were initiated. After 2 weeks, the affected arm was supported and flexed to 90°, then swung anteriorly, posteriorly, and laterally under the protection of the healthy arm (1–2 min/d until 4–6 weeks after surgery, then 1–2 min twice/d thereafter). Passive activities could be performed for shoulder abduction, flexion, and lateral external rotation training. Independent active activities were permitted at 12 weeks after surgery. An ice compress was applied for 20 minutes after rehabilitation training, 2 to 3 times/d. All rehabilitation training was completed under the guidance of the same doctor.

### 2.5 Statistical analysis

The SPSS software (ver. 23.0; IBM Corporation, Armonk, NY) was used for statistical analysis. The chi-squared test was used to analyze the injury classification data. The paired *t* test was used to compare the preoperative and postoperative measurement data within groups. The independent-sample *t* test was used for comparison between the LLP and BCP groups and for subgroup comparison. The results are expressed as means ± standard deviations. The significance level was set at *P* < .05.

## 3 Results

No significant difference in baseline characteristics (age, sex, disease course, injury type, ASES score, UCLASS, VAS score, flexion, abduction, or active lateral external rotation) was observed between the LLP and BCP groups (Table 1). No postoperative complication, such as infection, fever, neurological dysfunction, or joint adhesion, occurred. Pain and function scores, the active flexion angle, and subjective satisfaction scores were better in the LLP group than in the BCP group (all *P* < .05; Table 2). The operation time was shorter in the LLP group than in the BCP group (*P* < .05; Table 3). Among patients with good UCLASSs, the duration of preoperative pain was longer in the LLP group (*P* < .05); among those with acceptable UCLASSs, this duration was longer in the BCP group (*P* < .05). Among patients with excellent and good UCLASSs, the angle did not differ between groups; the preoperative flexion angle differed among patients with acceptable UCLASSs (*P* < .05). The preoperative external rotation angle did not differ between groups among patients with excellent UCLASSs. However, among patients whose results were acceptable, the preoperative frontal external rotation angle was greater in the LLP group than in the BCP group (*P* < .05; Table 4). No patient in the LLP group had a poor UCLASS, preventing comparison of this subgroup. Overall, the number of excellent UCLASSs was greater in the LLP group than in the BCP group (*P* < .05). The LLP group contained more small, medium, and large tear cases than did the BCP group (all *P* < .05). The total UCLASS, VAS score, and flexion, abduction, and body lateral rotation angle were greater in the LLP group than in the BCP group (all *P* < .05; Table 5). Patient sex, flexion strength, recovery time, the number of patients with excellent results of partial rotator cuff tear repair, and the ASES score did not differ between groups. Within the excellent, good, and acceptable UCLASS subgroups, age and the preoperative abduction angle did not differ between the BCP and LLP groups. The duration of pain also did not differ between groups.

**Table 1:** Patient characteristics and shoulder scores according to surgical position.

	Gender			Injury type (example)					
Group	M F	Age, yr	Disease course, mo	Part	Medium small	Large	ASES	UCLASS	VAS
LLP	65 50	63.4 ± 12.2	11.2 ± 1.6	23	72	20	48.4 ± 5.2	13.7 ± 1.7	6.1 ± 1.6
BCP	58 43	62.1 ± 13.4	12.4 ± 1.3	22	61	18	49.1 ± 4.3	14.1 ± 1.2	6.3 ± 1.5
Test statistics	0.018	0.451	1.300	0.937			–0.421	1.832	0.416
*P*	.893	.739	.315	.130			.671	.148	.741

ASES = American Shoulder and Elbow Surgeons; UCLASS = University of California at Los Angeles shoulder score; VAS = visual analog scale.

**Table 2:** Injury characteristics according to surgical position.

Group	Preoperative	Last follow-up
LLP		
Pain	3.8 ± 2.1	8.3 ± 3.7^a^^,^^b^
Function	4.1 ± 2.6	8.0 ± 3.3^a^^,^^b^
Active flexion angle	2.9 ± 1.8	4.6 ± 3.4^a^^,^^b^
Forward strength	2.1 ± 2.4	4.3 ± 3.2^b^
Subjective satisfaction	1.9 ± 2.6	4.6 ± 3.1^a^^,^^b^
BCP		
Pain	3.9 ± 1.9	7.5 ± 3.6^b^
Function	4.0 ± 2.5	7.3 ± 3.5^b^
Active flexion angle	2.8 ± 2.1	3.8 ± 3.2^b^
Forward strength	2.2 ± 2.5	4.1 ± 3.0^b^
Subjective satisfaction	2.0 ± 2.4	4.2 ± 3.2^b^

Comparison between the 2 groups at the last follow-up. BCP = beach chair position.

a *P* < .5; comparison between the same group before operation and the last follow-up.

b *P* < .5.

**Table 3:** Operation and recovery times and shoulder scores at last follow-up according to surgical position.

Group	Operation time, min	Recovery time, wk	ASES	UCLASS	VAS
LLP	81.4 ± 14.2	14.3 ± 5.1	93.0 ± 4.1	29.3 ± 2.1	0.7 ± 0.6
BCP	87.2 ± 15.5	14.8 ± 4.9	89.1 ± 5.3	26.1 ± 2.5	1.2 ± 0.8
Test statistics	2.091	–0.872	–0.982	28.713	0.763
*P*	.045	.064	.347	.036	.012

ASES = American Shoulder and Elbow Surgeons; UCLASS = University of California at Los Angeles shoulder score; VAS = visual analog scale.

**Table 4:** Patient and injury characteristics in UCLASS subgroups.

	Excellent		Good		Acceptable		Poor	
Group	LLP	BCP	LLP	BCP	LLP	BCP	LLP	BCP
sex								
M	51 (23.61%)	41 (18.98%)	4 (1.86%)	8 (3.70%)	1 (0.46%)	5 (2.31%)	0 (0%)	3 (1.38%)
F	48 (22.22%)	22 (10.19%)	7 (3.24%)	11 (5.09%)	4 (1.86%)	7 ( (3.24%)	0 (0%)	4 (1.86%)
Age, y	57.1 ± 12.3	58.9 ± 14.4	57.1 ± 12.1	56.3 ± 13.7	65.3 ± 15.1	67.5 ± 14.7	0 ± 0	68.3 ± 14.5
Pain time, mo	10.1 ± 1.3	11.2 ± 1.7	12.4 ± 1.5	9.9 ± 1.8	10.7 ± 1.8	14.5 ± 1.5	0 ± 0	14.3 ± 1.6
Preoperative flexion, °	65.1 ± 4.51	63.1 ± 4.62	48.3 ± 5.12	50.6 ± 4.25	42.7 ± 5.10	48.1 ± 4.32	0 ± 0	34.3 ± 3.35
Preoperative outreach, °	68.3 ± 5.13	71.3 ± 6.14	52.1 ± 7.21	50.2 ± 7.16	41.8 ± 7.63	40.6 ± 8.28	0 ± 0	36.5 ± 7.51
Preoperative external	13.5 ± 3.17	13.8 ± 3.25	6.3 ± 2.17	5.8 ± 3.16	5.9 ± 2.58	6.1 ± 2.37	0 ± 0	5.2 ± 2.64
Lateral rotation, °								
Type of injury part	19 (8.80%)	13 (6.02%)	3 (1.39%)	7 (3.24%)	1 (0.46%)	2 (0.93%)	0 (0%)	0 (0%)
Medium	65 (30.09%)	45 (20.83%)	5 (2.31%)	6 (2.78%)	2 (0.93%)	6 (2.78%)	0 (0%)	4 (1.86%)
Large	15 (6.94%)	5 (2.31%)	3 (1.39%)	6 (2.78%)	2 (0.93%)	4 (1.86%)	0 (0%)	3 (1.39%)

UCLASS = University of California at Los Angeles shoulder score.

**Table 5:** Functional ability according to surgical position.

	Forward bend, °				Outreach, °				Body lateral rotation, °			
Group	Preoperative	Last follow-up	*t*	*P*	Preoperative	Last follow-up	*t*	*P*	Preoperative	Last follow-up	*t*	*P*
LLP 53.24%	50.1 ± 4.23	160.3 ± 3.43	52.624	.000	55.3 ± 6.12	166.5 ± 6.12	53.513	.000	8.4 ± 2.41	57.5 ± 5.41	45.862	.000
BCP 46.76%	52.2 ± 4.31	153.5 ± 4.10	43.256	.000	54.7 ± 7.30	159.5 ± 7.31	49. 135	.000	8.5 ± 2.53	53.5 ± 5.71	42.351	.000
*t*	–0.452	0.892			–0.327	0.930			–0.281	0.879		
*P*	.651	.041			.782	.046			.729	.031		

BCP = beach chair position, LLP = lateral-lying position.

## 4 Discussion

This study is the first to compare the postoperative effects and factors influencing the arthroscopic treatment of rotator cuff injuries with patients in the LLP and BCP. Overall, we found that the postoperative effects of shoulder arthroscopy for rotator cuff repair performed with patients in the LLP were better than when the operation was performed with patients in the BCP. Patient sex and the preoperative abduction angle were not influencing factors that should be considered in posture selection for this procedure. Both positions are appropriate for the treatment of patients with partial rotator cuff tears and longer pain durations. The LLP is more suitable for those with small and large tears and large preoperative external rotation angles. The BCP is more suitable for patients with larger flexion angles.

Several factors may explain the higher total UCLASS and VAS score in the LLP group in this study. First, the use of the LLP with continuous upper-limb traction allows clear exposure of the surgical field and facilitates the identification of damage not seen on preoperative images,^[10]^ which contributes to comprehensive diagnosis and treatment. Second, the LLP allows surgeons to rotate the elbow for accurate positioning of the rotator cuff footprint area, and to directly visualize the depth of anchor screw insertion and apply sutures during rotator cuff repair. Many surgeons are able to perform operations with patients in the LLP and prefer to use this approach.^[11]^ In addition, obese patients can be managed more easily in the LLP than in the BCP, and brain desaturation events are rare when patients are in this position.^[12,13]^ The use of the LLP reduces the risk of cerebral thrombosis in patients with hyperlipidemia.^[14]^ In addition, the LLP better exposes the glenohumeral joint and facilitates location of the outer cortical area of the greater humeral tubercle.^[15]^ Finally, the LLP provides better intraoperative visualization due to the occurrence of less blistering, which facilitates the use of a posterior approach.^[11,15,16]^

Overall, excellent UCLASSs were more prevalent in the LLP group than in the BCP group in this study. No difference was observed between groups among patients with partial tears. The LLP was more advantageous than the BCP for the treatment of small, medium, and huge tears. At the last follow-up examination, the flexion, abduction, and external rotation angles were superior in the LLP group relative to the BCP group. These results are likely related to the several factors. The use of the LLP reduces tissue damage around the shoulder sleeve, thereby reducing postoperative adhesion, due to the clear exposure provided.^[15]^ The shorter operation duration achieved with the use of the LLP also contributes to the reduction of tissue damage. In addition, patients in this study did not have anterior shoulder instability, in contrast to the results reported by Frank et al.^[17]^ This result is attributable to the accuracy of diagnoses and the operators’ rich surgical experience and careful operation.

In recent years, postural change has been demonstrated to be more convenient when the patient is in the BCP relative to the LLP; an assistant can easily rotate the elbow to allow a surgeon to operate. The semi-upright nature of the BCP results in major hemodynamic changes in the brain. The LLP provides sufficient visualization to better expose the subacromial space and joint side of the rotator cuff,^[18]^ but the continuous upper-limb traction and brachial plexus traction carry a risk of injury.

As the equipment cost for beach chair positioning is significantly greater than that for lateral positioning,^[15]^ the financial burden on the patient should also be considered when choosing a patient position for shoulder arthroscopy. This study was conducted with a large sample and focused on rehabilitation effects, which allowed us to draw objective conclusions. However, this study was retrospective, rather than randomized and controlled. In addition, subjective scores, rather than MRI findings, were compared. Depending on patients’ wishes and economic situations, preoperative and postoperative MRI examination is recommended, as it provides objective data.

In conclusion, 2 positions can be used for the arthroscopic treatment of partial tears of the shoulder sleeve. Surgeons should choose positions suitable for individual cases to avoid complications as much as possible. In general, the LLP is more favorable. The LLP is more appropriate for small, medium-sized, and huge tears and patients with large preoperative external rotation angles, whereas the BCP is more appropriate in cases of greater preoperative flexion angles.

## Author contributions

**Investigation:** Daohua Chen, Dongfeng Chen.

**Visualization:** Rong Wu.

**Writing – original draft:** Minghua Zhang.

**Writing – review & editing:** Jiajing Lai.
